# Brief **CURRICULUM VITAE** - 2011

**Published:** 2012

**Figure:**
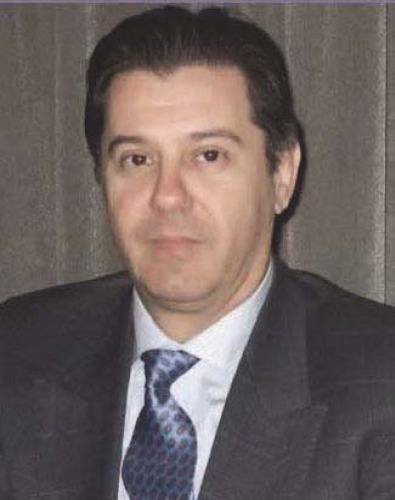


**Dr. Gelu Onose** - **54** years ; graduated, in 1982, from the Faculty of General Medicine, within the (former) Institute of Medicine and Pharmacy, in Bucharest

- **Professor** at the (State) University of Medicine and Pharmacy (UMF) “Carol Davila”, in Bucharest, Romania - the M6 Chair

- **MD**; **- PhD**; **- MSc**

- **Senior Physician** of : - Physical & Rehabilitation Medicine (PRM) and

- Gerontology & Geriatrics (G-G)

• **Chief of the M6 Chair** of the “**C. Davila**” **UMF** and of the **PRM Discipline** and of the **Physical and** (neural-muscular) **Rehabilitation Clinic Division** of the UMF, at the Teaching Emergency Hospital “Bagdasar-Arseni” (TEHBA), in Bucharest
• **President/ Co-Founder** of the **Romanian Society for Neurorehabilitation** (**RoSNeRa**) - affiliated to the World Federation for NeuroRehabilitation (**WFNR**) - member of its Management Committee - and respectively, of the **Romanian Society for Spinal Cord Pathology, Therapy and Rehabilitation in SCI** (**RoSCoS**) - affiliated to the Internationa Spinal Cord Society (**ISCoS**) and to the European Spinal Cord Injury Federation (**ESCIF**)
• A **member of the Scientific Committee**, **afferent to the Prezidium of the World Academy for Multidisciplinary Neurotraumatology** (**AMN**)
• **Selected and invited by Thomson Reuters**, **to participate in the invitation-only Academic Reputation Survey**”, within its related partnership with **Times Higher Education**'**s influential World University Rankings**.(2010 and 2011)
• **Invitated Peer**-**Reviewer** (in 2010) by the “**Journal of Molecular Histology**” (ISI Thomson Reuters rated)
• A member of the Board of the Romanian Society of Physical and Rehabilitation Medicine
• **8 published medical books** - one of them : “The Spondyloarthropathies”, and received, in 2002, the “Iuliu Hatieganu” Award of The Romanian Academy)
• **2 chapters within medical books**
• About 200 scientific works and papers - communicated within national and international scientific meetings and/or published in peer-reviewed or non peer-reviewed medical journals - and professional interviews/ articles, in mass-media
• **3 Invention Certificates, appointed by the State Office for Inventions and Marks**
• Main awards: the “Iuliu Hatieganu” Award of The Romanian Academy (2002); the Award of the (Romanian) National Authority for Scientific Research; the **Gold Medal at the International Saloon of Inventions**, **Geneva/ Switzerland** (2008)
• A **member of the Editorial Board** of medical journals:
• “Journal of Medicine and Life” (rated in PubMed, Index Medicus, Medline)
• “Infomedica”
• (Romanian) “Rehabilitation, Physical Medicine and Balneology”
• “Romanian Neurosurgery”
• **Main co-organizer** of the (biennial) **National Conference of Neurosurgery and NeuroRehabilitation, with International participation**

